# Association of delivery mode and number of pregnancies with anorectal manometry data in patients with postpartum constipation

**DOI:** 10.1186/s12884-023-05480-1

**Published:** 2023-03-11

**Authors:** Yan Yin, Yumin Zhang, Cheng Qian

**Affiliations:** General surgery department, Huzhou Maternity and Child Health Care Hospital, 313000 Huzhou, China

**Keywords:** Postpartum period, Parturition, Constipation, Anal sphincter, Manometry

## Abstract

**Objective:**

To explore the association of delivery mode and the number of pregnancies with anorectal manometry data in patients with postpartum constipation.

**Methods:**

This retrospective study included women with postpartum constipation treated at the pelvic floor rehabilitation department of Huzhou Maternity & Child Health Care Hospital between January 2018 and December 2019.

**Results:**

Among 127 patients included, 55 (43.3%) had one pregnancy, 72 (56.7%) had two pregnancies, 96 (75.6%) delivered spontaneously, 25 (16.7%) underwent Cesarean section, and six (4.7%) needed a Cesarean section despite spontaneous labor. The median duration of constipation was 12 months (range, 6–12). There were no differences between the two groups for any manometry parameters (all *P* > 0.05). The patients with a spontaneous delivery had a lower change in maximal contracting sphincter pressure compared with those with Cesarean section (14.3 (4.5–25.0) vs. 19.6 (13.4–40.0), *P* = 0.023). Only the delivery mode (Cesarean vs. spontaneous) independently affected the changes in contracting sphincter pressure (B = 10.32, 95%CI: 2.95–17.69, *P* = 0.006); age (*P* = 0.201), number of pregnancies (*P* = 0.190), and constipation duration (*P* = 0.161) were not associated.

**Conclusion:**

The patients with a spontaneous delivery had a lower change in maximal contracting sphincter pressure compared with those with a Cesarean section, suggesting that patients with Cesarean may retain a better “push” function during defecation.

**Study Highlights**:

1. WHAT IS KNOWN.

Changes in anal manometry are involved in constipation, and delivery affects the pelvic floor.

2) WHAT IS NEW HERE.

Patients with Cesarean section have higher changes in contracting sphincter pressure than vaginal delivery.

## Introduction

The reported prevalence of postpartum constipation is 45.8% [1]. Postpartum constipation may be the result of the high progesterone levels of pregnancy present during the early postpartum period, interruption of dietary intake and hydration during labor, use of analgesic or opiates during labor, perineal, episiotomy-related, or cesarean section-related pain, hemorrhoids, damage to the levator ani muscles and pelvic floor disorders, magnesium sulfate treatment, and cultural practices and dietary restrictions during the postpartum period [1]. In addition, prophylactic treatment with senna might increase the likelihood of having bowel movements within 24 h after childbirth [1]. Unfortunately, there is a lack of trials and studies on interventions and knowledge about postpartum constipation [2].

The anal sphincter generates pressure that can be measured using manometric techniques (e.g., water-perfused catheters, transducers, or balloons) [3–5]. Normal ranges obtained vary according to sex, age, and technique [3–5]. Sensations of the rectum and anal canal can be tested using balloon distension or electrical stimulation [4, 6]. These techniques can also provide thresholds for sensory urgency and maximal tolerable volume [4, 6].

Since constipation might involve pelvic muscle floor dyssynergia [7] and since pregnancy and delivery can damage pelvic floor function [8, 9], pelvic floor muscle training (PFMT) could play a role in the prevention and treatment of postpartum constipation. PFMT and biofeedback can be provided by a specialized nurse or a physical therapist. PFMT aims at maximizing strength and improving the coordination of the contractions during defecation [10]. PFMT is well-studied for urinary incontinence, but fewer data are available for fecal problems [11]. Nevertheless, available data suggest that PFMT can help improve the symptoms of dyssynergic defecation [12–14].

Still, the exact impacts of the delivery mode and the numbers of pregnancies on anorectal manometry are mostly unknown. Such data are needed to be able to monitor the changes during PFMT. Therefore, this study aimed to explore the association of delivery mode and the number of pregnancies with anorectal manometry data in patients with postpartum constipation.

## Methods

### Study design and patients

This retrospective study included women with postpartum constipation treated at the pelvic floor rehabilitation department of Huzhou Maternity & Child Health Care Hospital between January 2018 and December 2019. This study was approved by the Ethics Committee of Huzhou Maternity & Child Health Care Hospital. As a retrospective study, the requirement for informed consent was waived.

Constipation was diagnosed according to the Rome Criteria IV. The inclusion criteria were 1) with at least two of the following symptoms: (a) straining more than 25% of the defecations, (b) lumpy or hard stools of more than 25% of the defecations, (c) sensation of incomplete evacuation of more than 25% of the defecations, (d) sensation of anorectal obstruction/blockage more than 25% of the defecations, (e) manual maneuvers to facilitate more than 25% of the defecations, and (f) fewer than three spontaneous bowel movements per week, 2) hardly with loose stools if not using laxatives, 3) not meeting the irritable bowel syndrome diagnostic criteria, 4) underwent blood routine examination, blood examinations for thyroid functions, blood glucose, and blood calcium examinations to rule out relevant gastrointestinal diseases, and 5) no history of gastrointestinal diseases or relevant surgeries, and with no endocrine diseases. All patients did not use antibiotics, crude fiber, polysaccharides, probiotics, or other micro-ecological drugs. The exclusion criteria were (1) perianal diseases or relevant surgery history, (2) severe cardiovascular, cerebrovascular, or endocrine diseases, or (3) psychological diseases and could not cooperate with the examinations.

### Data collection

Demographic data of the patients (including age, number of pregnancies, delivery mode, and disease duration) were collected from the medical record system of the hospital. Data from the cough test, perception threshold, maximum volume, tolerance, and compliance were also collected.

The institution has strict examination preparation that is routinely applied for anorectal manometry. (1) The patient has to be free of serious perianal diseases such as hemorrhoids and anal fissures recently. (2) The patient is instructed to defecate at least 4 h before the examination and to lie down quietly for 5 min before the examination. (3) The patient is fully informed of the whole examination process and problems that might be encountered so that patients have full mental preparation. (4) If a spasm occurs during the test, the test is interrupted and tried again after a rest of 1 h.

The routine examinations for constipation include an evaluation using an 8-channel water-perfusion system (GAP 08 A) to measure the anorectal motility and anorectal pressure, including rectal dilatation threshold volume induced (rectoanal inhibitory reflex (RAIR)), rest pressure and maximal pressure of the anal canal, and length of the high-pressure zone.

### Statistical analysis

SPSS 26.0 (IBM, Armonk, NY, USA) was used for statistical analysis. Categorical data were described as *n* (%) and analyzed using Fisher’s exact test. The Shapiro-Wilk test was used for the normality test of the continuous data, showing that the data were not in normal distribution; therefore, the median (quartile) was used for description, and the Mann-Whitney U-test was used for comparison. The Spearman correlation test was used to assess the correlations between variables. A multivariable linear regression test was used to investigate the influencing factors of anorectal manometry data. Two-sided P-values < 0.05 were considered statistically significant.

## Results

### Characteristics of the patients

Table 1 shows the characteristics of the 127 patients. The median age was 30 years (range, 27–33). Among the patients, 55 (43.3%) had one pregnancy, and 72 (56.7%) had two pregnancies. In addition, 96 (75.6%) delivered spontaneously, 25 (16.7%) underwent Cesarean section, and six (4.7%) needed a Cesarean section despite spontaneous labor. The median duration of constipation was 12 months (range, 6–12).

**Table 1:** Characteristics of the patients

Characteristics	*n* = 127
Age, median (range)	30 (27–33)
Number of pregnancies, *n* (%)	
1	55 (43.3)
≥2	72 (56.7)
Delivery mode, *n* (%)	
Spontaneous delivery	96 (75.6)
Caesarean section	25 (16.7)
Spontaneous delivery + caesarean section	6 (4.7)
Disease duration, median (range)	12 (6–12)

### Anorectal manometry

Table 2 shows the anorectal manometry parameters according to the number of deliveries. There were no differences between the two groups for any manometry parameters (all *P* > 0.05). Table 3 shows the anorectal manometry parameters according to the types of delivery. The patients with a spontaneous delivery had a lower change in maximal contracting sphincter pressure compared with those with Cesarean section (14.3 (4.5–25.0) vs. 19.6 (13.4–40.0), *P* = 0.023).

**Table 2:** Comparison of anorectal manometry data in patients of different parities

Characteristics, median (range)	Parity 1 (*n* = 55)	Parity ≥ 2 (*n* = 72)	*P*
Rest pressure of the rectum	26.2 (16.1–43.9)	31.1 (20.7–49.4)	0.133
Rest pressure of the anal sphincter	84.3 (66.6-101.8)	90.6 (64.1-108.6)	0.576
Length of the anal sphincter	2.3 (1.6–2.7)	2.3 (1.4–2.5)	0.234
Effective length of the anal sphincter	1.8 (0.9–2.3)	1.5 (0.5-2.0)	0.120
First sensation volume	20.0 (15.0–20.0)	20.0 (15.0–20.0)	0.556
First threshold for the desire to defecate	40.0 (30.0–60.0)	40.0 (40.0–60.0)	0.923
Threshold of defecate distress	90.0 (60.0-120.0)	90.0 (60.0-120.0)	0.875
Perception threshold of the maximum volume	140.0 (115.0-180.0)	130.0 (120.0-180.0)	0.802
Rectal compliance	3.8 (2.1–7.3)	4.0 (2.1–8.6)	0.829
Relaxation rate of the anal sphincter	8.7 (0.0-23.1)	5.8 (0.0-20.5)	0.208
Pressure gradient of the rectum-anal sphincter	-42.5 (-67.2–17.2)	-42.5 (-66.1–15.3)	0.932
Maximum squeeze press (MSP)	106.0 (79.3-125.4)	108.8 (83.9–141.0)	0.203
Change in contracting sphincter pressure	16.3 (6.7–25.8)	15.2 (4.5–27.3)	0.990

**Table 3:** Comparison of anorectal manometry data in patients with different delivery modes*

Characteristics, median (range)	Spontaneous delivery (*n* = 97)	Caesarean delivery (*n* = 30)	*P*
Rest pressure of the rectum	27.4 (19.3, 47.8)	29.6 (18.8, 44.7)	0.923
Rest pressure of the anal sphincter	87.8 (64.3, 105.5)	84.0 (64.7, 102.1)	0.712
Length of the anal sphincter	2.3 (1.6, 2.7)	2.2 (1.3, 2.7)	0.585
Effective length of the anal sphincter	1.4 (0.8, 2.2)	1.6 (0.9, 2.1)	0.849
First sensation volume	20.0 (15.0, 20.0)	20.0 (15.0, 20.0)	0.906
First threshold for the desire to defecate	40.0 (30.0, 60.0)	50.0 (30.0, 60.0)	0.398
Threshold of defecate distress	90.0 (60.0, 120.0)	110.0 (60.0, 120.0)	0.409
Perception threshold of the maximum volume	120.0 (115.0, 180.0)	160.0 (120.0, 180.0)	0.238
Rectal compliance	4.0 (2.1, 7.6)	3.7 (2.4, 9.6)	0.865
Relaxation rate of the anal sphincter	6.2 (0.0, 20.0)	10.3 (0.0, 21.6)	0.393
Pressure gradient of the rectum-anal sphincter	-40.7 (-67.4, -16.6)	-49.3 (-59.6, -4.0)	0.867
Maximum squeeze press (MSP)	104.7 (80.3, 130.7)	121.1 (98.2, 142.5)	0.133
Change in contracting sphincter pressure	14.3 (4.5, 25.0)	19.6 (13.4, 40.0)	0.023

* Group according to the most recent delivery mode

### Factors influencing the changes in contracting sphincter pressure

Only the delivery mode (Cesarean vs. spontaneous) independently affected the changes in maximal contracting sphincter pressure (B = 10.32, 95%CI: 2.95–17.69, *P* = 0.006); age (*P* = 0.201), number of pregnancies (*P* = 0.190), and constipation duration (*P* = 0.161) were not associated (Table 4).

**Table 4:** Influencing factors of changes in contracting sphincter pressure

Characteristics	B	95%CI	*P*
Age	-0.57	(-1.44, 0.31)	0.201
Number of pregnancy (≥ 2 vs. 1)	5.19	(-2.62, 13.0)	0.190
Delivery mode (Caesarean delivery vs. spontaneous delivery	10.32	(2.95, 17.69)	0.006
Disease duration	0.78	(-0.31, 1.86)	0.161

## Discussion

This study aimed to analyze the anorectal manometry data of patients with postpartum constipation, to investigate the characteristics of the rectal pressure in patients with postpartum constipation, and to explore whether the mode of delivery, the number of pregnancies, and the postpartum time have an impact on postpartum constipation. The results suggest that the patients with a spontaneous delivery had a lower change in contracting sphincter pressure compared with those with Cesarean section, suggesting that patients with Cesarean retain a better “push” function during defecation. The results help provide a basis for better management of postpartum women and how to avoid post-delivery constipation in the future.

Understanding the physiology of the anal sphincter and anorectal pressure is important to improve the management of patients with dysfunctional defecation. Indeed, the anal sphincter maintains the anal function of the normal human body and controls the contraction and relaxation of the anus, including the external anal sphincter and internal anal sphincter [15]. The physiological function of the internal anal sphincter is mainly to close the anus and assist defecation. It is usually in a contractive state. The anus is closed to prevent feces, liquid, and gas from flowing out of the rectum and maintains a certain tension in the rectum [15–19]. This contractive state is not easy to fatigue, except for continuous work during defecation. When the rectum is filled with feces, it opens automatically to help defecate. The external anal sphincter has the function of encircling the anus. The external anal sphincter is composed of three U-shaped rings, making the anal canal tight. When the sense of defecation is generated, if the external conditions do not allow defecation, the anus can be closed, and defecation can be controlled by contracting the external sphincter [15–19]. However, the external sphincter is easy to fatigue, and the continuous contraction can only last for 55 s. After this time, the defecation will not be controlled and will be discharged from the body [19].

The rectum is another important structure involved in defecation. In the quiet state, the rectum is generally empty and collapsed. At that time, the resting pressure is about 0.49 kPa, and there are about five peristaltic waves per minute. When the amount of feces entering the rectum at one time reaches 10 ml, and the speed is fast, it will trigger the rectal dilatation threshold volume induced: the vertical contraction of the external sphincter and the puborectal muscle suddenly increases the anal pressure, which only lasts for 1 to 2 s. Then, the tension of the internal sphincter decreases slightly, and the anal pressure decreases slightly. After a few seconds, it can return to normal [18, 20]. When the content entering the rectum increases by about 220 ml and the internal rectal pressure reaches 4.61 kPa, not only does the internal sphincter already lose its self-control function, but also the strong urge to defecate and the characteristic that the continuous contraction of the pelvic floor muscle and the external sphincter is difficult to exceed 60 s will make the pelvic floor muscle and the external sphincter completely relax, and the anal pressure will fall together. At the same time, the internal rectal pressure will rise sharply due to the rise of the reflex abdominal pressure, up to 14.7 kPa, and the defecation power will exceed the defecation resistance. Then, rectal contents are excreted [18, 20]. Therefore, reasonable defecation includes the synchronous relaxation of the internal and external sphincters and pelvic floor muscles, an effective increase of defecation pressure, and unobstructed defecation channels. The reactive pressure rise of the anal canal to rectal expansion is called the anorectal constriction reflex, which represents the reflex and stress self-control function of the external sphincter. The anorectal suppression reflex refers to the automatic rise of anal pressure when the rectum dilates. It represents the reflex relaxation of the internal sphincter when the rectum is filled to facilitate discharge and the autonomic self-control function. Therefore, better pressure will also generate better impetus [18, 20].

Therefore, the present study examined the anorectal manometry parameters in women after delivery and the factors possibly influencing these parameters. The present study suggests that the number of pregnancies does not influence the anorectal manometry parameters. In addition, the only difference between patients with spontaneous delivery vs. Cesarean was a higher change in maximal contracting sphincter pressure than in patients with a cesarean. This higher value suggests that the patients with Cesarean retained a higher push strength and anorectal coordination during defecation [21, 22], as supported by Jordan et al. [23], who reported lower squeeze pressures at 3 months postpartum in women who delivered vaginally compared with Cesarean section. Chahila et al. [24] reported the changes in anorectal manometry before and after delivery but did not have a comparator group. Pregnancy causes strain to the pelvic floor [8, 9], and vaginal delivery is well known to be associated with injury to the pelvic floor [25–27], resulting in various postpartum conditions. The most common of these conditions are urinary and fecal incontinence [28, 29]. Still, damage to specific muscles can lead to pelvic muscle floor dyssynergia or decreased coordination [7]. A meta-analysis indicated a higher risk of fecal incontinence with vaginal delivery but highlighted that no analysis could be done for constipation because of the lack of a common assessment tool [30].

Available data suggest that PFMT can help improve the symptoms of dyssynergic defecation [12–14]. In addition, the present study suggests that patients who underwent cesarean section retained a stronger defecation strength than those who delivered vaginally, but how it could be used to personalize PFMT remains to be investigated. Of note, changes in anorectal manometry do not necessarily translate into anorectal dysfunction, and about 10% of women with postpartum sphincter defects are asymptomatic [4, 24, 31]. During PFMT, the therapist tries to maximize strength and improve the coordination of the contractions during defecation [10]. PFMT is well-studied for urinary incontinence, but fecal incontinence and constipation are also issues in many women after delivery and should be studied [11]. Constipation can result from many factors, including a low-fiber diet, dehydration, caffeine abuse, alcohol overuse, medications, endocrine disorders, neurological disease, and psychological issues [1, 32]. Physiological causes of constipation after delivery can include anal stenosis, anal stricture, abnormal musculature, and intestinal nerve abnormality [1, 32]. The results of the present study help us understand what occurs at the rectum level after delivery and could ultimately help optimize the PFMT for fecal issues, especially constipation.

This study has limitations. The sample size was small because the patients were from a single center. No control group was included since the patients were all from a department for pelvic floor rehabilitation. The retrospective nature of the study limited the data to those available in the patient charts. Among others, whether the patient displayed dyssynergia or whether the test had to be interrupted and retried could not be determined. The present study was retrospective, and only the anorectal manometry data could be analyzed. Causal relationships could not be determined. The actual meaning of the changes in anorectal manometry after delivery and the differences in manometry between modes of delivery in relation to the development of postpartum constipation and how to intervene on these changes remain to be investigated. This study was preliminary, and the sample size will be increased in the future. A prospective study is currently under preparation to investigate the dynamic changes in anorectal manometry after delivery and the impact of PFMT on manometry.

## Conclusion

The results suggest that the patients with a spontaneous delivery had a lower change in maximal contracting sphincter pressure compared with those with Cesarean section. The clinical significance of this weaker defecation push strength and how it fits within PFMT remain to be determined.

## Data Availability

All data generated or analyzed during this study are included in this article.

## Abbreviations

PFMT: pelvic floor muscle training
GAP 08A: 8-channel water-perfusion system
RAIR: rectoanal inhibitory reflex
